# Periocular Capillary Hemangiomas: Indications and Options for Treatment

**DOI:** 10.4103/0974-9233.63071

**Published:** 2010

**Authors:** Genie M. Bang, Pete Setabutr

**Affiliations:** University of Illinois Eye and Ear Infirmary, Department of Ophthalmology and Visual Sciences, University of Illinois at Chicago, Chicago, IL, USA

**Keywords:** Capillary Hemangioma, Eyelid, Orbit, Amblyopia, Corticosteroids, Kasabach-Merritt Syndrome, Propranolol

## Abstract

Capillary hemangiomas are the most common periocular and orbital tumors of childhood that typically arise in infancy. Though the diagnosis is frequently made on clinical examination, various diagnostic modalities may be helpful in initial evaluation and follow-up. Tests may be necessary in diagnosing suspect cases or aid in the differentiation of potential malignant tumors. In the vast majority of cases these tumors undergo spontaneous involution without sequelae. However, some periocular and orbital capillary hemangiomas require intervention to prevent serious complications. Other tumors require treatment to lessen the surgical burden for cosmetic repair. When treatment is necessary, there are a number of therapeutic options available. As there is no standard, potential risks and benefits must be discussed with the family and treatment should be specific in each case. A complete understanding of the natural history of the tumor, indications for treatment, and response to different therapies is imperative in managing this common lesion.

## INTRODUCTION

Capillary hemangiomas are the most common eyelid and orbital tumors of childhood. This tumor has been referred by many names (infantile hemangioma, juvenile hemangioma, hemangioblastoma, benign hemangioendothelioma, hypertrophic hemangioma), but is most commonly called ‘capillary hemangioma.’ This term is most common as it accurately describes the capillary unit structure of endothelial cells surrounded by pericytes.^1^,^2^

These tumors can present as small isolated lesions, or large masses that can cause visual impairment, systemic effects, or exist as part of a syndrome. Many capillary hemangiomas can be diagnosed on examination, but occasionally identification may require the use of ultrasonography, computed tomography or magnetic resonance imaging for accurate diagnosis. Understanding of this tumor, its natural history, and the indications for treatment are necessary for management of the tumor and to help the patient's family understand the disease process.

## EPIDEMIOLOGY

Capillary hemangiomas are among the most common tumors in infants, and the most common eyelid and orbital tumors of childhood.^1^,^3^ Approximately 1 in 10 children are affected by capillary hemangiomas of varying severity.^4^ However, histologic series do not reflect this high prevalence, as many hemangiomas are clinically diagnosed, and do not require biopsy. They typically occur as solid lesions, but up to 20 percent of affected infants have multiple lesions.^3^ Historically, females are three times as likely as males to be affected. Capillary hemangiomas are more frequent in premature or low-birth-weight infants.^1^ There is a large increase, approximately 10-fold, in infants born to women who underwent chorionic-villus sampling during pregnancy.^3^ There does not seem to be an association between capillary hemangiomas and race, ethnicity, or geographic location. There is no clear hereditary pattern or increased incidence among siblings. One-third of all eyelid and orbital capillary hemangiomas are diagnosed at the time of birth, and almost all are identified by six months of age.^1^

## NATURAL HISTORY

The exact etiology of capillary hemangiomas remains unclear, but likely involves a combination of angiogenesis and vasculogenesis. The natural history of capillary hemangiomas has been well documented over the past century. The tendency for these tumors to spontaneously involute is well accepted. Capillary hemangiomas typically present during the first few months of life, with 33% to 55% present at birth.^3^–^6^ Some of the early hemangiomas may present with ‘herald spots’, which are tiny red macular spots created by telangectatic vessels in the skin's superficial layers. Haik *et al* classified hemangiomas into three categories based on the color of the skin associated with the hemangioma: classic superficial strawberry nevus; subcutaneous hemangiomas that appear bluish or purple through the overlying, unaffected skin; and deep orbital tumors that present with proptosis without observable skin discoloration.^6^ Rootman, however, described a different classification, categorizing hemangiomas by level of skin involvement: strawberry nevus confined to the superficial dermis; superficial hemangiomas combined with subcutaneous involvement; and combined subcutaneous and deep orbital involvement.^7^ Capillary hemangiomas typically undergo the ‘proliferative phase’, of rapid growth within 3-6 months after initial appearance. Duration of this proliferative phase is variable, but usually occurs during the first year of life. The lesion then undergoes stabilization and involution. This involution typically begins centrally before extending to the periphery. Most of the involution occurs by age four to seven years, but has been noted to continue until the end of the first decade.^1^,^8^ Some superficial capillary hemangiomas may leave some skin change after involution, including fibro-fatty residuum or skin changes, which are often cosmetically concerning. Approximately 20 to 40 percent of patients will have residual changes in the skin after involution is complete.^3^ Early studies reported almost all patients were observed to show nearly complete resolution of the original hemangioma.^9^ However, it is important to note that these historical papers observed the natural history of capillary hemangiomas with minimal intervention and despite a significantly high spontaneous regression rate, the large periocular lesions frequently caused functional defects such as amblyopia, strabismus, and optic atrophy.

## CLINICAL PRESENTATION

Haik *et al* described a case series in which 25% had the classic strawberry nevus and a subcutaneous mass, 68% had the bluish-purple, ‘spongy’ subcutaneous mass, and 7% had no skin discoloration or visibly notable mass, but had deep orbital involvement.^6^ These deep tumors without superficial evidence often require further diagnostic testing for identification. As mentioned previously, Haik^6^ and Rootman^7^ described different classifications of capillary hemangiomas. These categorizations of hemangiomas are not mutually exclusive, as capillary hemangiomas can present in any combination in the superficial, subcutaneous, or deep orbital tissues. Haik also noted that 60% of the orbital capillary hemangiomas were found in the superior orbit, 16% in the inferior orbit, and 2% involving both upper and lower eyelids.^6^ Of the 101 patients in the case series, 38 had some degree of proptosis, ranging from mild (<2.0 mm) to severe (15 mm).^6^ Disfigurement, corneal exposure, and optic nerve compression can all occur, and may frequently worsen during the rapid proliferative phase.

Enlargement of the capillary hemangiomas can often be observed when the patients cry. Crying simulates the valsalva maneuver, which raises intrathoracic pressure, and theoretically forces deoxygenated blood into the capillary spaces of the hemangioma causing enlargement and deepening of the color. Haik's series noted this change in 46 of the 101 patients, so although this sign can be observed, it is not pathognomonic for capillary hemangiomas.^6^

## AMBLYOPIA

The most common ocular complication of periocular capillary hemangiomas in infants is visual loss secondary to amblyopia. Case reviews have reported between 40 to 60 percent incidence of amblyopia with periocular capillary hemangiomas.^6^,^8^,^10^ Vision loss can be due to strabmismus, anisometropia, or visual deprivation. Strabismus can occur from extraocular muscle infiltration or mechanical effect of the tumor. Indentation of the highly elastic infant sclera and cornea can induce high cylinder, resulting in anisometropic amblyopia, the most common type of amblyopia caused by periorbital hemangiomas. Astigmatic refractive errors have been reported in 20 to 46 percent of eyes with periocular capillary hemangiomas.^11^ The more severe deprivation amblyopia may develop when the tumor infiltrates the eyelid and obscures the visual axis. Astigmatism may be present more often in cases with upper eyelid involvement. Astigmatism may be permanent in some cases of the tumor with upper eyelid involvement. Robb concluded that total eyelid occlusion causing deprivation amblyopia, in concert with asymmetric refractive errors, resulted in the most severe amblyopia.^10^ Stigmar concluded the longer the visual axis was occluded, the more severe the amblyopia, reporting that 4 of 51 patients developed moderate to severe amblyopia after only 1 month of occlusion.^8^

The strikingly high incidence of amblyopia in some periocular infantile hemangiomas may be related to the onset of the tumor within the first few months of life. In numerous studies, it has been shown that young children may be susceptible to visual deprivation amblyopia in the first 7 years of life, though the sensitivity is likely greatest in the early months of life. Animal research models demonstrate severe amblyopia after only 1-4 weeks of visual axis occlusion.^1^

Previous studies have debated whether there is proven change in refractive error with treatment of the hemangioma. Robb suggested that there was no influence on refractive error by treating hemangiomas.^10^ Other studies have shown similar visual outcomes in patients who underwent conservative amblyopia treatment with glasses and patching, versus those patients who underwent hemangioma treatment such as steroid injection along with amblyopia treatment.^12^ Schwartz *et al* demonstrated there was a decrease in the amount of anisometropic astigmatism with reduction in the size of the capillary hemangioma.^11^ However they did note that a minority of the treated patients may have residual amblyopia, especially when the treatment began after 6 months of age. Schwartz *et al*, performed the largest review of periorbital capillary hemangiomas and studied the risk factors for amblyopia.^13^ They suggested that when evaluating a periocular capillary hemangioma, size greater than 1 cm in diameter is an important predictor of amblyogenesis, and approximately half of these patients developed amblyopia. PHACES syndrome (Posterior fossa malformations, Hemangiomas, Arterial anomalies, Cardiac defects and coarctation of the aorta, Eye abnormalities, and Sternal abnormalities or ventral developmental defects) is a rare neurocutaneous syndrome that has been noted to have severe hemangiomas, including periocular hemangiomas. Diffuse hemangiomas and those associated with PHACES syndrome are more likely to cause amblyopia.^14^

## SYSTEMIC ASSOCIATIONS

Capillary hemangiomas have been associated with hematologic, cardiac, respiratory and infectious disorders. Large hemangiomas are subject to superficial ulceration, and may rarely cause significant hemorrhage or become secondarily infected with resultant sepsis.^1^

Kasabach-Merrit syndrome is a rare, but feared hematologic abnormality associated with extensive capillary hemangiomas. This disorder was first described in 1940, and is characterized by platelet and/or fibrinogen entrapment and consumption coagulopathy. Numerous cases have been reported, however it is rarely associated with isolated facial hemangiomas. In Kasabach-Merrit syndrome, a critical thrombocytopenia occurs from platelet sequestration within the tumor's vascular channels. Clinically, thrombocytopenic purpura is visible, along with large ecchymoses around the hemangioma or elsewhere on the body. Kasabach-Merrit syndrome can lead to sudden, massive hemorrhage with resultant cardiovascular collapse. Treatment of this syndrome consists of systemic steroids and thrombocyte-replacement therapy, with a 50% response rate. Recalcitrant cases can be further treated with radiotherapy, surgical removal or embolization, however this syndrome has a high mortality rate.^1^,^3^ Consumptive coagulopathies associated with capillary hemangiomas can manifest with consumption of clotting factors, increased bleeding time, decreased levels of factors V and VII, low fibrinogen, and increased fibrolysis.

Microangiopathic hemolytic anemia (MAHA), has also been described with large capillary hemangiomas of the periocular region, digestive tract, and the liver. In MAHA, the erythrocytes are destroyed from coagulation, or are sheared or fragmented by high pressure forcing them through the abnormally small vessels of the hemangioma.^1^ High-output congestive cardiac failure may occur in patients with large visceral hemangiomas, as the abnormal arteriovenous communication causes a ‘short circuit’ in the systemic circulation. Arterial blood is then shunted through a systemic vein because it is the ‘path of least resistance’, and a resultant increase in overall peripheral resistance occurs. Therefore, cardiac output must increase to continue perfusion of the distal vasculature, and if the infant heart cannot compensate with dilatation, tachycardia, and cardiac hypertrophy, then congestive heart failure may occur.^1^ This complication is often fatal, but rarely occurs with isolated cutaneous hemangiomas. It is more often reported in concert with visceral hemangiomas of the liver, lungs, heart, intestines, spleen and bones. Thus, it is important to complete a full evaluation of an infant, and to rule out visceral involvement when evaluating cutaneous hemangiomas, as visceral involvement may carry 40% to 80 % morbidity and mortality.^3^

Periocular capillary hemangiomas may occur simultaneously with oral, nasal, subglottic, and paratracheal hemangiomas. These hemangiomas can pose a threat by compressing the adjacent trachea or larynx, thus causing respiratory distress and asphyxiation, or can compress the esophagus, leading to secondary aspiration. There may be a risk from uncontrolled bleeding from these hemangiomas, leading to potential aspiration or massive hemorrhage.^15^ The hemangiomas seen in PHACES syndrome are associated with an increased risk of amblyopia and more likely to require treatment.^15^

## DIAGNOSTIC TESTING

While capillary hemangiomas can largely be diagnosed through clinical examination, some tumors are not easily distinguished. The differential diagnosis includes rhabdomyosarcoma, lymphangioma, chloroma, neuroblastoma, orbital cysts, and cellulitis, hence additional diagnostic testing may be necessary.

### Plain radiography

Plain radiography is not particularly helpful in the diagnosis of capillary hemangiomas. These tumors are not easily seen or clearly identified on plain film. Soft tissue enlargement can be seen, but bony structures will not show evidence of bony erosion. If there is rapid growth of the orbital tumor during development, orbital enlargement can be seen. However, none of these findings are diagnostic for capillary hemangioma.

### Ultrasonography

Ultrasonography scan may show an irregular mass with variable reflectivity which blends into surrounding orbital structures. With the use of A-scan ultrasonography, areas of low internal reflectivity, moderate reflectivity, and high reflectivity may be identified. The low areas represent the areas of solid, hypercellular endothelial proliferation, whereas the moderate echoes represent ecstatic vascular channels, and the high reflectivity corresponds to fibrous septae separating the tumor lobules. Orbital involvement and monitoring of tumor size can be accomplished with the use of ultrasonography.^1^,^2^

### Computed tomography

Computed tomography (CT) is another modality for monitoring tumor growth. Capillary hemangiomas often appear as homogeneous soft tissue masses of the orbit, without destruction of orbital bone. The tissue mass occurs in the anterior orbit, or in the extraconal space with ‘finger-like’ posterior extension. This mass can be well defined or irregular and infiltrating the surrounding tissue.^1^ With the use of contrast material, the capillary hemangiomas may enhance, aiding in the identification of the border. The feeder vessels may also be evident with the use of contrast. CT imaging can also be utilized to view normal orbital structures, and to evaluate the relationship of the tumor mass to the optic nerve, extraocular muscles, and other orbital structures.

### Magnetic resonance imaging

On Magnetic Resonance Imaging (MRI) studies, capillary hemangiomas may appear as well-circumscribed densely lobulated tumors. On T1-weighted MRI, the capillary hemangioma is of intermediate signal intensity, but it is moderately hyperintense on T2-weighted imaging due to the slow blood flow through the vascular channels. The tumor mass may enhance following gadolinium contrast injection, so images should have fat suppression on T1 weighted images to better visualize the tumor.^1^,^3^

### Angiography

Angiography is rarely used as a diagnostic tool for capillary hemangiomas, as both venography and arteriography are technically difficult, and not without complications. Arteriography is rarely used diagnostically, but may be useful in rare situations to identify feeder vessels for possible ligation or embolization in life-threatening hemangiomas that are non-responsive to less invasive therapies.^1^ Feeder vessels may be identified as an enlarged ophthalmic artery, internal maxillary artery, or frontal branch of the superior temporal artery. The tumor often drains into the cavernous sinus via the superior ophthalmic vein.

### Tissue biopsy

Although capillary hemangiomas can often be diagnosed with less invasive techniques, tissue biopsy may be occasionally required, especially in the case of proptosis without superficial involvement. In these cases, the differential diagnosis may include other tumors in this age group, such as lymphangioma, metastatic neuroblastoma, or rhabdomyosarcoma. As all surgeries carry inherent risks of infection, hemorrhage, diplopia, or visual loss, biopsy diagnosis is reserved for cases that cannot be diagnosed with less invasive techniques.

## THERAPY

### Indications for therapy

Indications for therapy of capillary hemangiomas are important to note, as the natural course for this tumor is spontaneous involution. Visual indications include occlusion of the visual axis, amblyogenic anisometropia, optic nerve compression, or significant proptosis causing exposure keratopathy. Dermatologic indications for treatment are maceration and erosion of the epidermis caused by severe hypertrophy of the epidermis and subcutaneous tissues. Systemic indications for treatment include: obstructive (nasopharyngeal, oral, or subglottic extension causing airway obstruction), hematologic (thrombocytopenia or hemolytic anemia), and cardiovascular (high-output congestive heart failure).^1^

Careful discussion with the family is imperative when contemplating any treatment. It is important to carefully describe the risks and benefits of each therapy, including vigilant monitoring, vascular occlusion, excision, radiation, intralesional or systemic corticosteroid, interferon, laser, or the newly introduced propranolol therapy.

### Amblyopia therapy

It has been well documented by several published reports that adenexal capillary hemangiomas can result in severe and permanent visual loss.^1^,^8^–^10^ Hemangiomas are usually present at birth, and tend to grow rapidly during the phase critical to visual development, hence there is a serious concern for deep amblyopia. It is critical to monitor these patients for any changes in fixation preference or signs of amblyopia, and to discern when to initiate interventions to treat hemangiomas. Visual acuity should be assessed with cycloplegic retinoscopy at least monthly until the hemangioma starts to regress, or signs of amblyopia, anisometropia, or strabismus become evident. When astigmatism of more than 1.50 to 2.00 D is present, the visual axis is obscured, or the lesion begins to expand rapidly, treatment of the tumor should be strongly considered. Additionally if there is evidence of amblyopia, the normal eye should be patched and optical correction, if needed, should be prescribed.^1^

Strabismus surgery is usually delayed until amblyopia therapy is completed, and the hemangioma shows signs of regression. In doing so, there is less potential for hemorrhagic complications, and improved predictability of surgical outcomes. Frequently, surgery is not necessary, as the strabismus often resolves as the tumor regresses.^1^

### Surgical options

Surgical removal is usually reserved for cases where more conservative therapy has failed, and tumor removal is necessary. The highly vascular tumor may create a significant higher risk of intraoperative bleeding. Surgery can reduce tumor size by compromising the tumor feeder vessels, constriction and sclerosing of the small vascular channels, or primary excision of the entire mass can be performed. Historically there have been attempts to stimulate the natural involution of capillary hemangiomas by destroying the small vascular channels and stimulating thrombosis through the use of compression, cryotherapy, diathermy, or injection of sclerosing agents or even boiling water. These techniques may accelerate the involutional process, but may also end in more pronounced cosmetic and functional deformities.^1^,^6^

Actual excision of the tumor is sometimes effective for tumors that are small and non-responsive to pharmacologic therapy, or remain after the natural involutional. Primary surgical excision during the proliferative phase, or for large tumors is not ideal, as these tumors tend to have greater infiltration, are less encapsulated, and carry higher risk of bleeding associated complications. If surgical excision is absolutely necessary, it is important to obtain preoperative imaging, including MRI, MRA, and possibly an angiogram in order to identify the vascular channels of the tumor. As these large hemangiomas can infiltrate surrounding structures, there is the complication of unintended excision of surrounding orbital structures. The surgeon must also be prepared for the possibility of considerable blood loss especially in pediatric cases with limited blood supply, hence blood-typing is necessary. Due to the potential for such severe complications, surgical excision is best utilized very early, with small lesions, or to repair remaining deformity after involution of the tumor.

### Radiotherapy

Radiation therapy can be effective in the management of certain capillary hemangiomas. Microembolisms can be created in the tumors by low-level radiation, which can speed the regression of the mass. Involutional response usually occurs in 1-2 weeks, and treatment may be repeated for further resolution. Cumulative doses must be monitored as historically, large doses of radiation with poor delivery or shielding could cause radiation-induced complications of the skin, eyelids, eye and orbit. Current radiologic technology makes direct radiologic damage rare, however radiation induced cataract can still occur with 200-600 rads if the anterior segment is not adequately shielded.^1^

### Laser Therapy

Carbon dioxide, argon, neodymium-YAG, and flash-lamp pumped-dye laser have all been utilized in the treatment of capillary hemangiomas.^1^,^3^ Laser treatments have been found to be more effective for early and superficial lesions and less effective in deeper orbital lesions.^1^,^3^,^16^ Some physicians have found more success with combination therapy, such as intralesional steroids in combination with laser therapy. There is not yet a defined laser-delivery system or method specific for the different types of hemangiomas and most physicians find results are user dependent.

### Systemic corticosteroids

Zarem and Edgerton first reported the use of systemic corticosteroids in the successful treatment of large capillary hemangiomas of seven infants in 1967.^17^ This treatment has been used frequently with variable success. The most commonly used dose of oral corticosteroid is 1-2 mg/kg prednisone daily or 2-4 mg/kg every other day. Response has been variable, but regression is most often noted in the first 2 weeks of treatment. Some have noted poor response in orbital tumors, and also a high incidence of rebound growth with tapering or discontinuation of steroid therapy, prompting a longer duration of steroid treatment. This long duration of steroid treatment raises the concern of steroid treatment in infants, including growth delay, adrenal suppression, cushingoid features, hyperglycemia, delayed wound healing, arterial hypertension, hypertrophic cardiomyopathy, and susceptibility to infection.^1^,^18^

The exact mechanism of action of systemic corticosteroids is speculative. It has been proposed that steroids have an anti-anabolic effect on the immature vascular tissue of capillary hemangiomas.^1^,^17^ Others believe the mechanism of steroid action might be secondary to a vasoconstrictive effect, where the corticosteroids increase the vascular sensitivity to already circulating vasoconstrictive agents, thus causing constriction induced hypoxia and resultant regression. There is also theory of the pharmacologic inhibition of angiogenesis. In experimental models, corticosteroids have demonstrated reduction in the rate and extent of blood vessel growth.^19^ Corticosteroids may also inhibit activators of fibrinolysis in the vessel walls thereby increasing the tendency towards coagulation.

### Intralesional corticosteroid injection

Due to the systemic risks of long term oral steroid use, intralesional corticosteroid injection is more commonly used. A recent study of American Association for Pediatric Ophthalmology and Strabismus members, reported that intralesional steroid injection is the most common treatment and also the most frequent initial therapy.^16^ This modality allows for a high dose of medication directly into the tumor, minimizing systemic absorption. One or two additional injections are usually required after the initial injection. Intralesional corticosteroid injection was first described in non orbital cutaneous hemangiomas, and was reported for periocular lesions by Kushner in 1979. His case series reported success in 3 out of 4 patients with the use of triamcinolone and betamethasone.^20^ Injection can be used most easily with anterior lesions or after biopsy or partial excision of deeper orbital tumors. There have also been descriptions of techniques involving sub-Tenons injection along with injection of the hemangioma, with satisfactory results.^21^ For intralesional injection, Kushner recommends 40 mg of triamcinolone acetate and 6 mg preparation of betamethasone acetate and betamethasone phosphate, with a total injection volume of 1-2 ml. The risk of potential systemic absorption from intralesional injection exists hence dosing based on patient weight is recommended. Injections can be separated in order to allow for multiple injection sites and facilitation of even distribution, although some clinicians advocate single site injection to theoretically reduce tissue pressure and retrograde flow.^22^ Corticosteroids should be injected under low injection pressure while the needle is slowly withdrawn. The tumor may enlarge from the medication and rarely bleed, requiring gentle pressure for hemostasis. Retinal vessels should be examined during and after injection to monitor for central retinal artery occlusion.

Complications from local corticosteroid therapy are reportedly low and apparently vary with frequency, quantity, and method of injection. The most serious and feared complication is central retinal artery occlusion (CRAO).^23^ It has been speculated that increased force from the injection or digital pressure after the procedure may cause retrograde flow of the steroid particles into the central retinal artery. Corticosteroid particles can be up to 8 times the size of erythrocytes. There has been a case report of bilateral retinal embolization after corticosteroid injection of a right periocular capillary hemangioma.^24^ Two days post-injection, the infant was observed to be visually inattentive by the parents, and examination revealed bilateral retinal embolization with visible corticosteroid particles in the fundus. The authors speculated that the tumor had feeder vessels from both ophthalmic arteries, and retrograde flow resulted in bilateral deposition of particles.

Other complications following intralesional corticosteroid injection include eyelid necrosis, subcutaneous deposits, focal hypopigmentation and fat atrophy. Transient adrenal suppression and cushingoid symptoms have been reported.^24^,^25^ This transient suppression emphasizes the significance of avoiding live attenuated virus vaccination for 4 weeks before and after injections.

### Interferon α

Recombinant interferon alfa, and angiogenesis inhibiting agent, has been used successfully as a secondary agent for life-threatening hemangiomas that have failed corticosteroid therapy.^1^,^18^ Both interferon alfa-2a and 2b have been used as subcutaneous injections. The dosing is usually 3 million units per square meter of body-surface area per day. Response to treatment usually ranges from a few weeks to several months. Some of the few common side effects include irritability, liver enzyme abnormalities and neutropenia. The most worrisome side effect is spastic diplegia, which has been reported in up to 20 percent of patients. The mechanism is not understood and is potentially irreversible.

### Vincristine

Due to the serious side effect of spastic diplegia, vincristine replaced Interferon α as the second-line treatment for large capillary hemangiomas resistant to corticosteroids. Vincristine is a microtubule inhibitor, and is also an immunosuppressant used in treating thrombotic thrombocytopenic purpura or chronic idiopathic thrombocytopenic purpura. It has be shown to be efficacious in the treatment of Kasabach-Merritt syndrome with non-orbital capillary hemangiomas.^26^ Side effects include irritability, loss of deep tendon reflexes, and abdominal pain. Intravenously infused vincristine necessitates the use of a permanent venous port in the patient, hence its use is reserved for capillary hemangiomas that are life threatening and unresponsive to other medications.

### Cyclophosphamide

The use of an alkylating agent for the treatment of capillary hemangioma was first reported by Rush in 1966, with subsequent case reports verifying the efficacy of cyclophosphamide for life-threatening and corticosteroid resistant hepatic hemangiomas.^27^ The regimens described were 10mg/kg per dose for 3 to 4 days intravenously or orally for 2 or 3 courses.^18^ Cyclophosphamide is an alkylating agent that is thought to block or decrease new capillary proliferation. Reported side effects range from none, to transient myelosuppression and elevated liver enzymes. Serious toxic effects of cyclophosphamide, such as gonadal damage, hemorrhagic cystitis, and secondary malignancies are considered unlikely with low dose and short term therapeutic use. Cyclophosphamide has not been reported as a single therapeutic agent for the treatment of capillary hemangioma and may be considered suitable adjunct therapy to avoid the toxicity of long-term interferon treatment.^18^

### Imiquimod

Imiquimod is an immune-response modifier that induces production of cytokines from keratinocytes and monocytes to cause regression of numerous dermatologic conditions, including warts, superficial basal cell carcinoma, squamous cell carcinoma *in situ*, and actinic keratosis. Imiquimod has been found successful in treating superficial capillary hemangiomas of the scalp, eyebrow and forehead. This is a new agent with future potential however, safety or efficacicy for ophthalmic use is still under investigation.^10^

### Systemic propranolol

Systemic propranolol is a new treatment modality introduced by Leaute-Labreze in 2008.^28^ Propranolol is a non-selective beta-blocker that has long been used in the pediatric population for cardiac indications. Leaute-Labreze *et al* serendipitously observed a dramatic decrease in the size of cutaneous capillary hemangiomas in an infant being treated with propranolol for a cardiac indication. In follow up report, they observed improvement or regression of the hemangiomas in 31 of 32 patients. One patient discontinued treatment because of wheezing.^28^ Propranolol is now being used successfully for laryngeal and periocular capillary hemangiomas.^4^,^28^,^29^ The suggested dosage of oral propranolol is 2 to 3 mg/kg/day, divided twice daily, and adjusted for weight changes during growth of the infant. Beta blockers do have the risk of exacerbating reactive airway disease, bradycardia, systemic hypotension, and masking of symptoms of hypoglycemia in the infant population. It is suggested that the patient undergo cardiac evaluation prior to treatment and monitoring of the blood pressure, heart rate and blood sugar during the first 24 hours of treatment. Authors have discussed that the masking of hypoglycemic symptoms is valid in patients who are premature or not feeding well but not as much of a concern in patients in patients who are healthy and feeding normally. Others suggest monitoring vital signs and glucose levels while titrating propranolol up to the final treatment dose.^30^ Wheezing is a relative indication for discontinuation of the treatment. The exact mechanism of non-selective beta-blockage on the regression of capillary hemangiomas is not completely understood at this time. Proposed mechanisms include vasoconstriction, decreased expression of vascular endothelial growth factor (VEGF), and induction of apoptosis of capillary endothelial cells.^12^,^31^,^32^ Due to the long history of use in the infant population, and the relatively low risk, some are advocating systemic propranolol as a first-line treatment for periocular and laryngeal hemangiomas that require treatment.^5^

## SUMMARY

Capillary hemangiomas are the most common tumors in infants, and the most common eyelid and orbital tumors of childhood. The diagnosis is usually made clinically, although other diagnostic modalities are occasionally employed. Most tumors undergo spontaneous involution, however, some tumors require intervention to prevent serious complications. Other tumors require treatment to decrease the surgical burden for cosmetic concerns. There are many treatment options available for the practicing clinicians, and there is no exact standard for these treatment modalities. More recently, systemic propranolol has been found to be safe and effective in the treatment of eyelid and orbital hemangiomas. A complete understanding of the natural history of the tumor, indications for treatment and response to different therapies is vital to properly treat these common lesions.
